# Nitrogen-fixing bacteria and *Oxalis* – evidence for a vertically inherited bacterial symbiosis

**DOI:** 10.1186/s12870-019-2049-7

**Published:** 2019-10-23

**Authors:** Michelle Jooste, Francois Roets, Guy F. Midgley, Kenneth C. Oberlander, Léanne L. Dreyer

**Affiliations:** 1 Department of Botany and Zoology, University of Stellenbosch, Private Bag X1, Matieland, 7602 South Africa; 2 Department Conservation Ecology and Entomology, University of Stellenbosch, Private Bag X1, Matieland, 7602 South Africa; 3 H. G. W. J. Schweickerdt Herbarium, Department of Plant and Soil Sciences, Plant Sciences Complex, University of Pretoria, Private Bag X20, Hatfield, 0028 South Africa

**Keywords:** *Bacillus*, Endophytic bacteria, Geophytes, Nitrogen fixation, Oxalotrophic bacteria, *Oxalis*, Vertical transmission

## Abstract

**Background:**

Plant-endophyte symbioses often revolve around nitrogen metabolism, and involve varying degrees of intimacy. Although evidence for vertical inheritance of nitrogen-fixing endophytic bacteria is increasing, it is confined mostly to crop plants, and to date no such system has been reported for geophytes.

**Methods:**

Bacterial endophytes associated with *Oxalis*, the most species-rich geophytic genus form the Cape Flora in southern Africa was studied. Culturable endophytes were isolated from surface-sterilized vegetative and reproductive plant organs for six host species at three locations. Colonies of microbes on various artificial media were morphotyped, enumerated and identified using sequence data. Filter exclusion experiments were conducted to determine if endophytes were vertically transmitted to seeds, determine if mucilage plays a role to actively attract microbes from the soil and to assess microbial richness isolated from the mucilage of *Oxalis* seedlings. Fluorescent microscopy was implemented in order to visualize endophytic bacteria in cryo-sectioned seeds.

**Results:**

Evidence for a novel, vertically transmitted symbiosis was reported. Communities of nitrogen-fixing and plant growth-promoting *Bacillus* endophytes were found to associate with selected *Oxalis* hosts from nitrogen-deficient environments of the Cape. *Bacillus* endophytes were ubiquitous and diverse across species and plant bodies, and were prominent in seeds. Three common nitrogen-fixing *Bacillus* have known oxalotrophic properties and appear to be housed inside specialised cavities (containing oxalates) within the plant body and seeds.

**Conclusions:**

The discovery of vertical transmission and potential benefits to both host and endophyte suggest a particularly tight mutualism in the *Oxalis*-endophyte system. This discovery suggests unexpected ways in which geophytes might avoid nitrogen deficiency, and suggest that such symbioses are more common than previously expected.

## Background

The Greater Cape Floristic Region biodiversity hotspot (Cape) of southern Africa is globally renowned for its diverse and extremely species-rich flora [1, 2]. To date at least some of this remarkable diversity has been attributed to abiotic factors such as palaeoclimatic stability, reliable seasonal water availability, geographical gradients and diverse soil types [3, 4]. The sandstone derivation and low pH of most soils, together with predictable winter rainfall and relatively frequent wildfires all contribute to dystrophic conditions with severe nitrogen deficiency [4]. These include some of the lowest nitrogen and phosphorus levels measured globally [5]. Nitrogen is essential to the growth and development of all terrestrial plants [6–8]. There is growing evidence that diverse Cape plant lineages have adapted by forming associations with growth promoting and nitrogen-fixing micro-organisms [9]. Recently it has been suggested that plant-microbial interactions play an important role in generating, shaping and maintaining ecosystem diversity within the Cape [10, 11]. Many of the most diverse and ecologically dominant indigenous Cape plant lineages form classical ‘textbook’ symbioses with beneficial micro-organisms [12–17]. Unfortunately, relatively little research attention has been given to these associations; it is evident that limited information is available addressing the mechanisms, diversity and role of microbial associations of Cape plants [18].

The Cape is also renowned for the most diverse geophyte flora in the world, including 2100 species from 20 families [19, 20]. Although the factors driving this remarkable Cape geophyte diversity are still poorly understood, geographical distribution, climatic factors (rainfall quantity and reliability) and plant growth form (storage organ size) have been suggested [21]. The role of plant-microbial interactions has, however, not yet been confirmed [22].

*Oxalis* is the largest geophyte genus in the Cape (180 spp.), and has undergone an extensive radiation (ca. 230 spp.) in southern Africa [1, 2] that likely originated in the Cape [23]. The evolutionary success of this genus in the Cape may partly be attributed to its unique life history, which includes a geophytic habit, winter flowering and variable seed strategies. However, the evolutionary success of this genus is still poorly understood. Cape *Oxalis* species are highly unusual in terms of their seed germination strategies as approximately 60% have recalcitrant seeds. These seeds germinate immediately when shed from fruits and display an inverted germination order compared to most angiosperms where foliar leaf development and growth followed by delayed radicle growth (Additional file 1: Figure S1). This remarkable germination strategy raises the question of how these seedlings are able to photosynthesize and grow without a well-developed radicle or roots. A set of observations that suggest that *Oxalis* has developed a unique association with nitrogen-fixing and/or growth promoting endophytic bacteria (EB) has been uncovered. Such associations may help explain the ecological (and evolutionary) success of *Oxalis* under challenging Cape edaphic conditions.

This study was focused on beneficial plant-microbial interactions and considered: 1) the presence of EB associated with Cape *Oxalis* species, 2) the EB community composition within hosts and between locations, and finally 3) elucidating the nature of relationships between host plants and endosymbionts.

## Results

### Bacterial species found in vegetative and reproductive organs

**Fig. 1:**
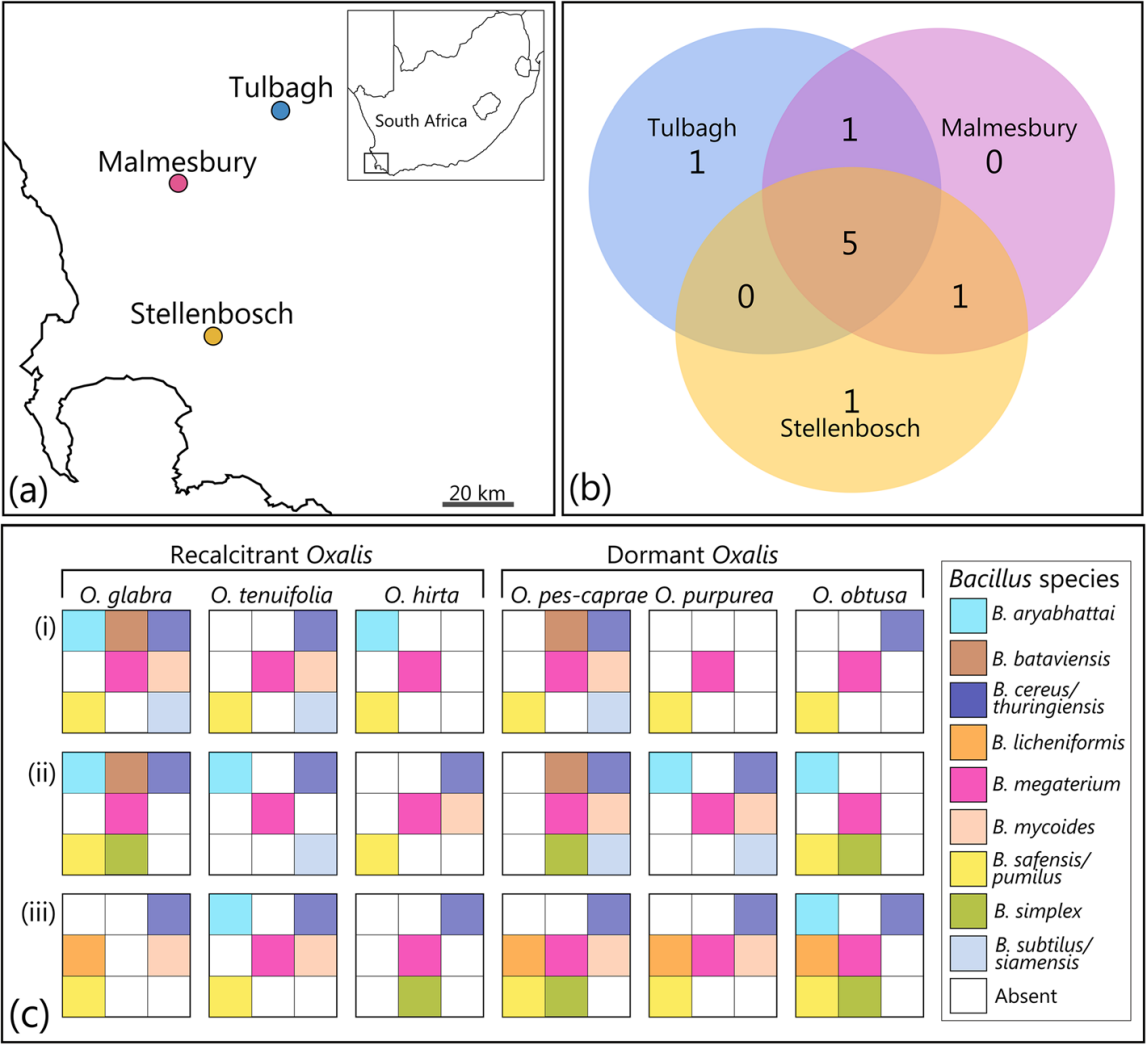
Community composition and diversity of the most abundant and consistent endophytic bacteria (EB) isolated from *Oxalis* hosts. **a** Sampling locations within the Cape of southern Africa, **b** Unique and shared EB diversity across sampling locations, **c** Endophytic *Bacillus* species isolated from reproductive and vegetative plant organs (combined isolates from roots, bulbs, leaves, stems and seeds) and five plant replicates of six *Oxalis* hosts sampled at three locations: (i) Tulbagh, (ii) Malmesbury, (iii) Stellenbosch

The presence and diversity of EB were assessed amongst vegetative and reproductive organs of six *Oxalis* host species sampled from three different locations (Fig. 1a). Pure-culture bacterial colonies isolated from sterilized, macerated plant material were identified using universal 16S bacterial primers. Sequencing revealed that 92% of replicates were consistent with morphotyping, with all three replicates showing similar 16S sequences with less than 2% base pair difference among sequences of approximately 800 base pairs. For the remaining two replicate sets, one sequence differed from the other two. This error margin (2.6% of morphotypes incorrectly assigned), is small enough not to substantially affect the conclusions of this study. Sequencing was used to identify bacterial colonies from seeds and morphotype records were used to determine the distribution of bacterial species throughout the remainder of plant organs that were not sequenced.

A labile community of cultivable EB were associated with *Oxalis* hosts, where nine of the most abundant and consistent EB species were from the genus *Bacillus.* These species included *B. aryabhattai* Shivaji et al. 2009*, B. bataviensis* Heyrman et al. 2004*, B. cereus/thuringiensis* Frankland and Frankland 1887/Berliner 1915*, B. licheniformis* Weigmann 1898*, B. megaterium* de Bary 1884*, B. mycoides* Flügge 1886*, B. safensis/pumilus* Satomi et al. 2006/Meyer and Gottheil 1901*, B. simplex* Priest et al. 1989 emend. Heyrman et al. 2005/Sumpavapol et al. 2010 and *B. subtilus/siamensis* Cohn 1872. The most likely bacterial species identifications were based on 16S sequences and phylogenetic comparison (Additional file 1: Figure S1).

Six bacterial endophytes were identified to species level and morphological traits (bacterial cell lengths) were used to distinguish between *B. megaterium* and *B. aryabhattai* that had unresolved relationships based on the consensus tree. There were three instances where it was not possible to distinguish between two closely related species. As these species are morphologically indistinguishable from one another, these endophytes were consequently referred to as *B. cereus/thuringiensis*, *B. safensis/pumilus* and *B. subtilus/siamensis*, indicating the two most likely identities of these isolates based on BLAST results. It should be noted that *B. cereus* and *B. subtilus* have previously been isolated from the rhizosphere and roots of two *Oxalis* species from Columbia [29] and were therefore considered as the most likely EB. More conclusive identification for these latter taxa requires additional markers.

*Bacillus* endophytes were identified from various host plant organs including roots, bulbs, stems, leaves and seeds, as well as the rhizosphere soil surrounding plant roots. Most (77.8%) of the EB sampled from plant tissues were also present in the rhizosphere of the specific host plant studied. This suggests that these EB are a subset of rhizosphere bacteria. It is possible that the remainder of EB reached plants through colonization events during previous growing seasons, vertical transmission from parent plants or failure to isolate or identify specific EB during culturing either due to isolation protocol or EB was outcompeted by other stains on agar media. The majority of EB (91.1%) occupied all sampled plant organs and all host species contained at least two EB species, across all recalcitrant and orthodox species. This indicated that these EB are generalist endophytes that are not host species-, organ- or germination strategy-specific. Sampling location may have influenced endophytic community composition, as one or two unique EB were isolated from each site or were shared among two sites (Fig. 1b). However, five out of the nine identified EB were shared among all three locations (Fig. 1c), indicating a universal association between *Bacillus* endophytes and *Oxalis* hosts.

It should be noted that seed bacterial diversity (*Bacillus* species) was a small subset (on average 25%) relative to the total bacterial and fungal morphotype diversity isolated per plant (roots, bulbs, stems and leaves). The true endophytic diversity of bacteria and fungi colonising *Oxalis* hosts may be much larger than reported in this study.

EB were isolated from surface-sterilized, macerated seeds from all host species and locations. Multiple pure-culture colonies isolated from the vegetative (roots, bulbs, leaves, stems) and seed tissues from a single host plant were sequenced using universal bacterial 16S primers. Among 88.9% of studied cases identical sequences of EB isolates were obtained from parent plant and seed material. Isolation and identification of these EB indicated that vertical transmission from parent plants to their offspring would most likely explain this observation.

### Filter exclusion experiments to assess horizontal and vertical transmission

Seeds and fruits from 230 angiosperm genera are known to excrete carbohydrate-rich mucilage around the seed coat and base of the hypocotyl [32]. Most recalcitrant Cape *Oxalis* species produce large amounts of mucilage upon germination. Filter exclusion experiments were conducted to assess if EB inhabit the mucilage of germinating seedlings, and to determine if additional microbes from the soil actively move towards the mucilage. Filters had an average pore size of 10-15 μm, while all identified EB had dimensions of 3.19 × 1.33 μm (length SD = 1.770 μm, width SD = 0.671 μm).

**Fig. 2:**
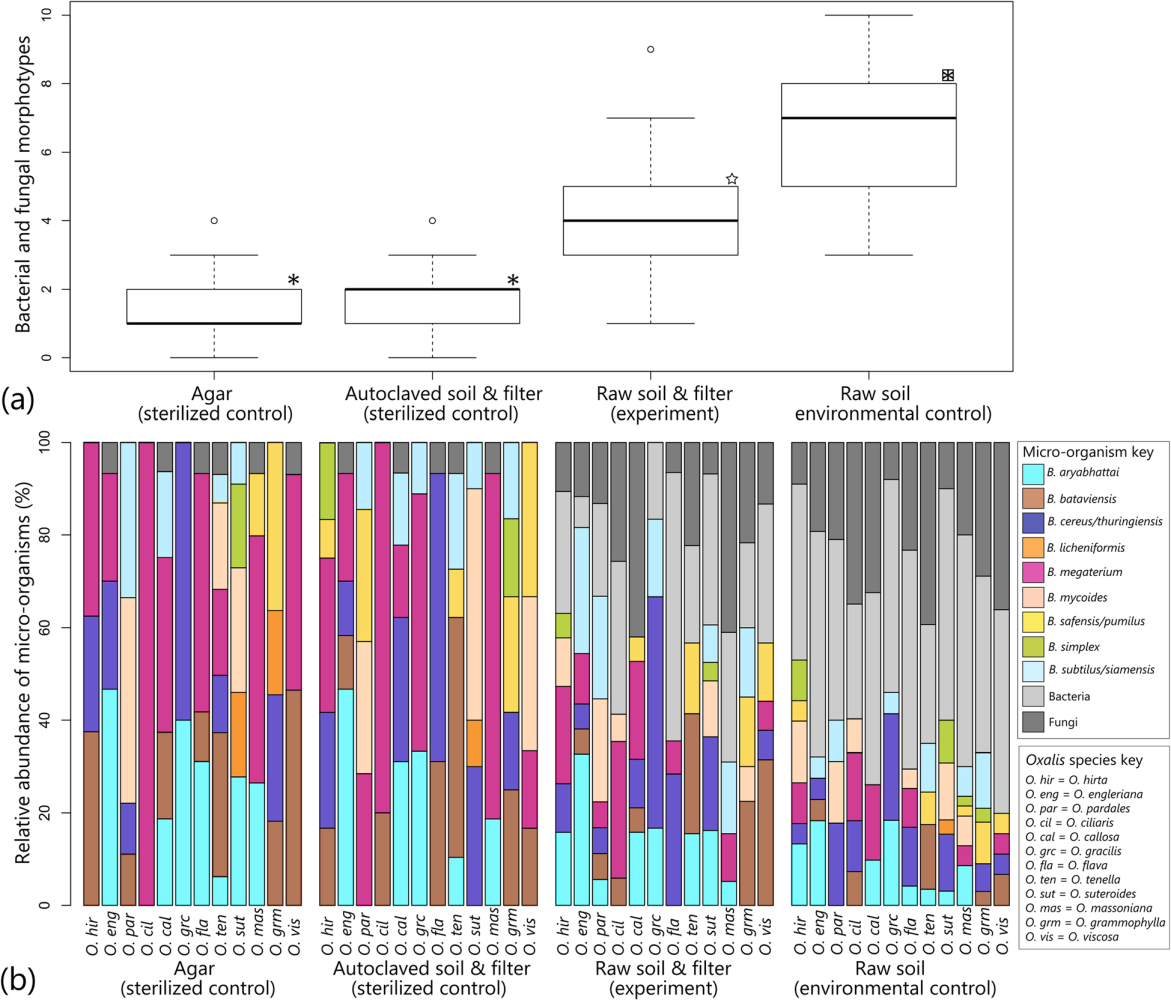
Abundance and community composition of micro-organisms (bacteria and fungi) isolated from mucilage produced by *Oxalis* seedlings. **a** Number of bacterial and fungal morphotypes isolated from the mucilage produced per seedling, averaged across 12 *Oxalis* species per treatment. All symbols are significantly different at *p* < 0.0001. **b** Diversity and relative abundance of bacterial and fungal morphotypes associated with *Oxalis* mucilage. Each vertical bar represents percentage of each bacterial/fungal morphotype isolated across five seedling replicates per *Oxalis* species

Morphologically similar colonies were identified with a success rate of 97.4%, with less than 2% base pair difference among sequences of approximately 800 base pairs. If morphologically new/unknown colonies were encountered they were given a unique morphotype number. Fungal isolates were documented and identified, but are not discussed in this article. Using the sequenced and identified EB from macerated plant material as reference material, this study has shown that the same suite of *Bacillus* species and no other cultivable bacteria were present in the mucilage of developing seedlings when germinated on sterile media (Fig. 2a–b). The mean number of species obtained from both negative control treatments were significantly greater than zero (sterile agar mean = 1.67, ±1.118 SE; sterile soil mean = 1.89, ±1.150 SE, z = 4.592, *p* > 0.0001), confirming the presence of seed endophytes that were vertically transmitted from parent plants to their offspring. There was no significant difference between the number of species from the two sterile negative control treatments (z = 0.908, *p* = 0.795). The total diversity of vertically transmitted EB associated with each *Oxalis* host species (i.e. total bacterial species diversity across all within-species replicates) was higher than the average values reported for individual seedlings per species. Different combinations of EB species associated with individual seedling replicates.

Environmental control treatments had the highest number of isolates per seedling (mean = 6.80, ±1.121 SE), while treatments with a filter had a significantly lower average number of isolates (mean = 4.08, ±1.129 SE) (Fig. 2a). Both the filter exclusion treatment and the environmental control had significantly higher microbial diversity (z = 7.399, *p* < 0.0001 and z = 12.343, *p* < 0.0001 respectively) relative to the negative controls. Sampling location may have had an effect on number of isolates, as the two *Oxalis* species sampled from the common garden collection had significantly fewer isolates per treatments (mean = 1.17, z = − 2.363, *p* = 0.018) than field-species. This may indicate a bottleneck effect in loss of microbial diversity in the garden soils or that unfavourable growth media were used to detect such EB if they were present. This experiment showed that a diverse assemblage of soil bacteria and fungi inhabit the mucilage of *Oxalis* seedlings, some which may be actively recruited from the rhizosphere and others provided (via vertical inheritance) by the seed itself (Fig. 2b).

### Potential plant growth promoting propertied if isolated EB

Among bacterial species isolated from macerated *Oxalis* seeds, 90.9% were identified as EB with well-documented growth promoting and/or nitrogen-fixing properties (Additional file 4: Table S1). This implies a possible role for selection favouring vertical transmission of beneficial endophytes. Three of the most prevalent endophytic species isolated from all *Oxalis* hosts and locations were the diazotrophic bacterial species *B. cereus/thuringiensis*, *B. megaterium* and *B. safensis/pumilus* (Fig. 1c). Symbiosis with diazotrophic and plant growth promoting EB can be considered a highly beneficial association from the host plant’s perspective. Preliminary δ^15^N data obtained from 83 *Oxalis* species illustrated a wide range of values (16.78 – 2.09^o^/_oo_), including relatively light values approaching the range reported for plants associated with N-fixing EB. However, due to limited sampling and lack of reference soil samples [33], this requires further testing. To our knowledge this is the first report of a potentially nitrogen-fixing association between EB and geophytes. Future research should explore the extent of N-fixation in both seedlings and mature plants.

Potential benefits to the EB associates have not been tested, but could include a carbon-source for energy and housing inside *Oxalis* plant tissues. To date, nine EB have been isolated from four *Oxalis* species globally [34], and seven of these have known oxalotrophic properties. These include *Azospirillum brasilense* Krieg & Döbereiner, 1978*, B. amyloliquefaciens* Priest et al. 1987*, B. cereus, B. subtilis, B. vallismortis* Roberts et al. 1996*, Methylobacterium oxalidis* Tani et al. 2012 and *Serratia fonticola* Gavini et al. 1979 [34, 35]. Oxalotrophic bacteria have the metabolic capacity to utilize oxalates as their only (and often preferred) carbon source [35, 36], and typically form symbioses in order to access these compounds. Oxalotrophic bacteria have been isolated from various habitats [37], but are most commonly found in the rhizosphere and roots of plants that excrete large amounts of oxalate. Oxalates most often occur in the form of oxalic acid or calcium oxalate crystals among members of the genus *Rumex* and *Oxalis* [37–39]. Due to the toxicity and low energy yield of oxalates, most microbes cannot utilize these as an energy source [35, 40].

## Discussion

In this study three EB with known oxalotrophic activity were ubiquitous among Cape *Oxalis*: *B. cereus/thuringiensis, B. licheniformis* and *B. subtilis*. Importantly all three of these oxalotrophs have been reported as nitrogen-fixers, and at least one of each was isolated from the seeds of every sampled host plant. These findings could indicate a strong association between *Oxalis* plants and diazotrophic oxalotrophic EB. Vertical transmission typically evolves when symbiotic associations are mutualistic, in order to ensure that the beneficial endosymbionts are transferred from one generation to the next [41, 42]. A recent study showed that oxalotrophic properties among EB were required to ensure colonization and transmission within host plants [43].

**Fig. 3:**
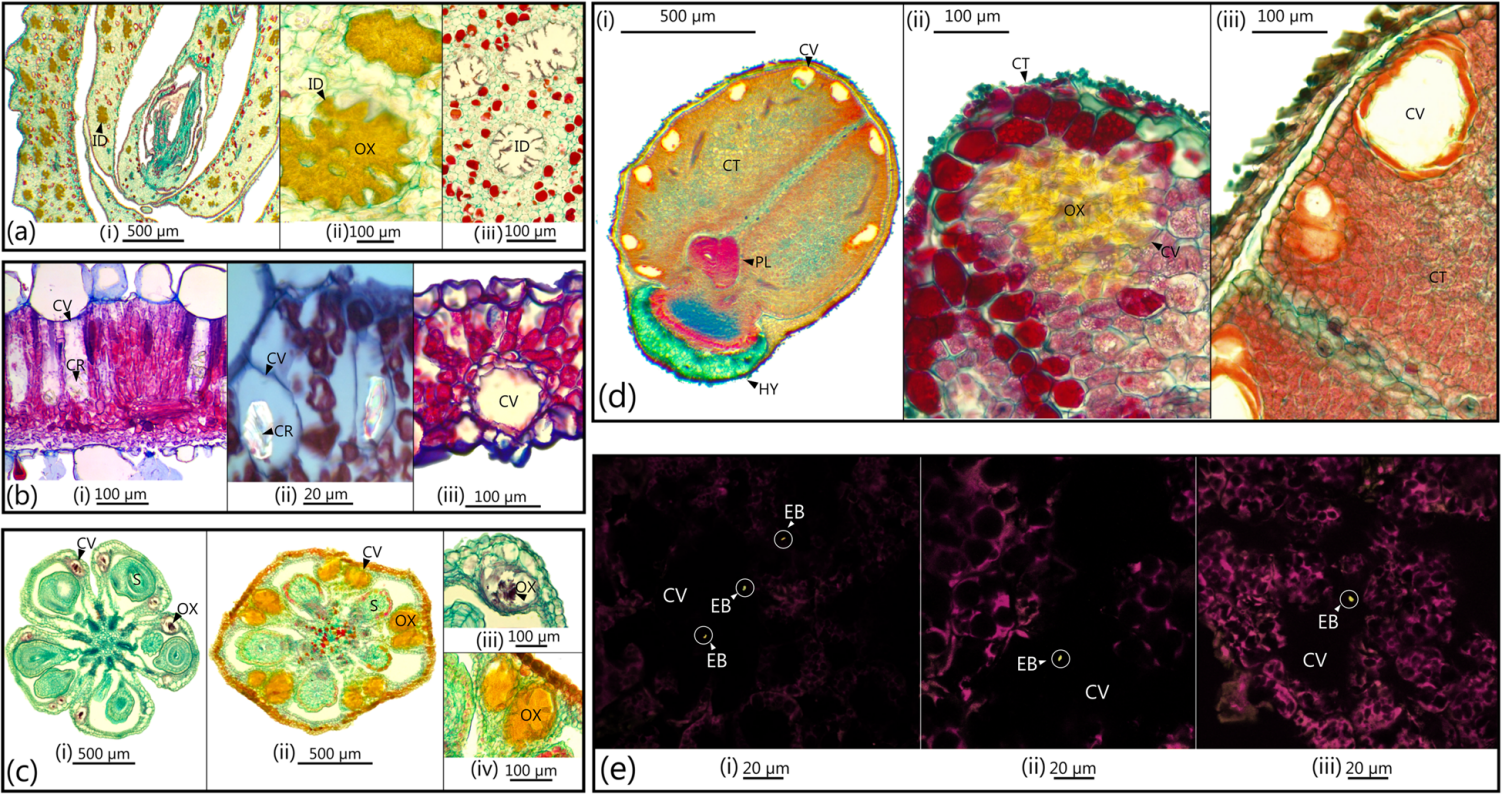
Specialized idioblast cells and cavities containing oxalates, and potential endophytic bacteria within Cape *Oxalis* host plants. **a** Longitudinal sections through bulb fleshy leaves with idioblasts containing oxalates (crystals) (i,ii) and a cross section of empty cavities (iii). **b** Cross sections of photosynthetic leaves with cavities containing oxalates (i,ii) and empty cavities with an epithelial lining (iii). **c** Cross sections of dormant (i) and recalcitrant (ii) fruit capsules with cavities containing oxalates. **d** Longitudinal sections of recalcitrant seeds with multiple cavities in the cotyledons (i), a cavity containing oxalates (ii) and empty cavities with epithelial lining (iii). **e** Confocal staining of cross sections of sterilized seeds indicating bacteria (bright green rods, circled in white) inside cavities. Original red-and-green confocal images supplied in Additional file 3: Figure S3. EB = endophytic bacteria, CO = cotyledon, CR = crystals, CV = cavities, HY = hypocotyl, ID = idioblasts or idioblast cavities, OX = oxalates, PL = plumule, S = seeds. A key to all species names is provided in Additional file 5: Table S2

Oxalates are by-products of photorespiration [44] and high oxalate concentrations could be harmful to plant tissues, especially the photosynthetic system [45]. To avoid such damage, plants often compartmentalize oxalates as deposits within intracellular (ordinary cells or specialized crystal idioblasts) or extracellular structures (cavities) [46, 47]. Crystal idioblasts and cavities harbouring calcium oxalate crystals are well-documented in storage, vegetative and reproductive tissues of *Oxalis* [38, 48], including Cape *Oxalis* (Fig. 3a–e). Given that idioblasts and cavities are common among *Oxalis* species, and that oxalotrophic nitrogen-fixing EB were ubiquitous in *Oxalis* hosts, it is possible that endophytes might be housed inside these structures. That *Oxalis*-associated oxalotrophic EB would utilize oxalates as a carbon source, and in turn supply the hosts with biologically fixed nitrogen, is thus an intriguing possibility.

In this study the majority (95.5%) of seeds with large and/or abundant idioblasts or cavities with oxalates were detected among embryo tissue of recalcitrant *Oxalis* species. It is possible that this structural adaptation, together with the mucilage production associated with germination, could help ensure that recalcitrant species host and transfer the beneficial endophytes to their seeds. Even though oxalotrophic diazotrophic EB were detected among all *Oxalis* species, it is likely that recalcitrant seedlings may be most reliant on these associations given their inverted germination sequence. Additional nitrogen could lessen the need for immediate root growth, which could allow a resource allocation shift from root germination to leaf growth instead. It is possible that an association with EB were a pre-adaptation to such an unusual inversed sequence of germination and rapid growth and establishment of seedlings.

If shown to have benefit to the host, such a widespread association with EB could be a key mechanism that allows *Oxalis* to thrive and diversify in such nutrient-depleted environments as those present in the Cape. The discovery of this symbiosis could hold critical implications for the current understanding of the distribution of *Oxalis*, its anatomy and physiology, the establishment of seedlings and evolution of this genus within the Cape Flora. Given the lack of data for endophytic associations in other geophytes, this may be a more widespread phenomenon in the Cape and globally. This knowledge is also directly relevant to all conservation efforts, invasion biology and predictions of the expected response of this genus and other Cape plants under current climate change predictions.

Most importantly, all of these EB strains were isolated from surface-sterilized *Oxalis* seeds, illustrating vertical transmission from parent plants to the next generation. To date, growth promoting and nitrogen-fixing seed endophytes have been recorded among well-studied crop plants [49, 50], but are rarely documented among wild plants [51]. The confirmation that nitrogen-fixing EB are vertically transmitted among geophytes indicates that it may be a far more widespread phenomenon than previously thought. To date the only other known examples of vertical transmission of diazotrophic bacteria occur in the giant cordon cactus (*Pachycereus pringlei S. Watson*) [52] and an invasive grass (*Sorghum halepense* L.) [53], both from North America.

The many beneficial traits associated with diazotrophic and/or oxalotrophic seed EB could be used for applications in natural and agricultural systems, especially for plants grown in harsh and nutrient-depleted conditions [54, 55]. *Bacillus* species are widely known to be safe and highly effective when used to enhance crop growth and yield [56]. The roles and importance of nitrogen-fixing EB capable of vertical transmission, represents a fascinating, yet largely understudied aspect of the Cape Flora.

## Conclusions

The discovery of vertical transmission of *Bacillus* endophytes from parent plants to the next generation and potential benefits to both host and endophyte, suggest a particularly tight mutualism in the *Oxalis*-endophyte system. Given that idioblasts and cavities are common among *Oxalis* species, and that oxalotrophic nitrogen-fixing EB were ubiquitous in *Oxalis* hosts, it is possible that endophytes might be housed inside these structures. This discovery also suggests unexpected ways in which geophytes might avoid nitrogen deficiency, and suggest that such symbioses are more common than previously expected.

## Methods

### *Oxalis* plant material and sterilization protocol

Six phylogenetically representative *Oxalis* species (*O. glabra* Thunb.*, O. hirta* L.*, O. obtusa* Jacq.*, O. pes-caprae* L.*, O. purpurea* L. and *O. tenuifolia* Jacq.) were sampled from three locations (Malmesbury [− 33.481121, 18.753625], Stellenbosch [− 33.932358, 18.874571] and Tulbagh [− 33.311688, 19.096747]) in the Western Cape Province, South Africa. All plants were correctly identified and harvested from the wild (research sample collection permit obtained from the Western Cape Nature Conservation Board, South Africa). Five individuals of each species (at least 10 m apart), with no external signs of microbial infections (asymptomatic), were collected at each location during May-June of 2016 and 2017. Plants were dug out with minimal disturbance to the below-ground organs and all excess soil was shaken off, until no soil was visible on roots and bulbs. Before samples were processed for endophyte isolation, plant roots were gently washed in 5 ml sterile water in order to obtain rhizosphere samples. Hereafter roots, bulbs, stems/rhizomes (depending on the above-ground growth forms of species: *O. glabra*, *O. hirta* and *O. tenuifolia* have above ground stems; *O. pes-caprae*, *O. purpurea* and the sampled populations of *O. obtusa* do not), leaves and seeds of all plants were aseptically separated using a scalpel and individually surface sterilized. For surface sterilization, samples were washed in a 33% dilution of household bleach (±5% sodium hypochlorite) for 1 min and 75% ethanol for 1 min, interspersed with three one-minute washes in sterile water. As sterilization controls a few additional samples of each of the plant organs were dabbed onto bacterial plate count agar (Biolab, Merck) and malt extract agar (Sigma-Aldrich), and incubated for 7 days at 28 °C in the dark - no colonies were detected.

### Isolation of bacterial colonies

Plant organs were manually cut into small segments using a scalpel and transferred into Eppendorf tubes (to the 0.5 mL mark) with five sterile glass beads and sterile water to 1.5 mL, under sterile conditions. Samples were macerated using a TissueLyser (Qiagen TissueLyser, Retsch MM301) at the Central Analytical Facility at Stellenbosch University. 200 μL each of 1:4 sterile-water-diluted macerate was plated onto three agar media: bacterial plate count agar (Biolab, Merck), nutrient broth agar (Sigma-Aldrich) emended with 4 g potassium oxalate (BMS Education) per litre of agar and malt extract agar (Sigma-Aldrich). Potassium oxalate was added to nutrient broth agar to re-create the high oxalate content of the host plant (as described by Sahin (2005); agar medium with pH 5.51). After 5 days of incubation at 28 °C in the dark, all morphologically different colonies (in terms of colour, shape, size and/or texture) were sub-cultured onto fresh plates. This process was repeated after another 5 days of incubation for each identified morphotype until pure cultures were obtained. All bacterial morphotypes were recorded and photographed. Three individuals of each morphotype per *Oxalis* species from each location were kept to test accuracy of morphotype identification. Each representative morphotype was divided, with 50% used for DNA extraction and sequencing, and the remainder stored in 50% glycerol in cryostorage (− 80 °C).

### DNA extraction, amplification and sequencing

To assess accuracy of endophyte morphotyping, three replicates from 25 different bacterial morphotypes were sequenced, with the expectation that the DNA sequences of each morphotype triplet would be identical. Morphotypes isolated from seeds and bulbs (reproductive propagules) were prioritized for sequencing in this study. DNA extractions were done following a modified 2X CTAB protocol, as described in Oberlander et al. [24]. Amplification and sequencing of the 16S rRNA region was conducted using universal bacterial primers 27F (5′-AGAGTTTGATCMTGGCTCAG-3′) and 1492R (5′-ACCTTGTTACGACTT-3′) [25]. PCR amplification reactions were performed using 25 μL reaction mixtures consisting of 9 μL dH2O, 2.5 μL MgCl_2_ (25 mM), 0.25 μL of each primer (10 μM), 12 μL KapaTAQ (KM1000, Kapa Biosystems) and 1 μL DNA (326 to 498 ng/μL). The PCR thermal cycling conditions were: 94 °C (5 min), 30 cycles of 94 °C (1 min), 49 °C (1 min) and 72 °C (2 min), with final extension at 72 °C (10 min). PCR products were sequenced at the Central Analytical Facility at Stellenbosch University. Confirmation of base calling in sequence chromatograms was manually conducted in Chromas v. 2.6.5 (http://www.technelysium.com.au). Sequences were aligned using the embedded ClustalW function [26] in BioEdit v. 7.2.6 [27]. Obtained sequences were compared to GenBank (NCBI) submissions using online BLAST searches.

### Phylogenetic analysis

Phylogenetic trees were reconstructed using a dataset of 16S sequences for endophytic bacteria (excluding duplicate sequences), sequences downloaded from Genbank (three representative BLAST results per *Oxalis* endophyte sequence) as well as four bacterial outgroup species. Tree samples were generated in MrBayes (v. 3.2) using the nst = mixed command for model selection and a gamma rate correction for 2 X 10^7^ generations under otherwise default settings. Convergence of parameter values was checked in Tracer (v.1.6) [28] 25% burnin. The consensus tree was used to aid species identification (Additional file 1: Figure S1).

### Filter exclusion experiments

The aim of these experiments was to determine if EB were transmitted from parents to seedlings (vertical transmission) and determine if mucilage plays a role to attract additional microbes from the soil (horizontal transmission). Filter exclusion experiments were conducted to assess microbial richness isolated from the mucilage of *Oxalis* seedlings. Sterilized gel drying frames (Sigma-Aldrich) were used as a filter barrier between the germinating seed and a selected treatment medium. The pore size (10-15 μm) of these filters was large enough to allow movement of microorganisms through the filter. Filters were wet with sterile water in order to prevent an artificial pulling-effect when seeds with mucilage were placed on the filters. Experimental filter treatments were compared to two negative controls (sterile conditions) and one positive environmental control (soil).

Mature seeds were harvested from 12 recalcitrant *Oxalis* species (20 seeds per species) that are known to produce large amounts of hypocotylar mucilage. For each species, a soil sample (10x10cm and 5 cm deep) was collected at each sampling location (10 species sampled from filed locations and 2 species sampled from a research collection at the Stellenbosch University Botanical gardens). A research sample collection permit was obtained from the Western Cape Nature Conservation Board, South Africa for all seeds harvested from the wild. Seeds were surface-sterilized according to the above-mentioned protocol. Seeds were randomly divided into four groups of five seeds each and assigned to one of four experimental treatments. Treatment 1 served as a negative control where seeds were placed on sterile agar. Treatment 2 served as an additional negative control where seeds were placed on a sterilized filter and a sample of autoclaved soil within a sterile petri-dish. Treatment 3 consisted of a sample of raw soil within a petri dish with a sterilized filter placed on top. Seeds were then placed on top of the filter to determine if microbes actively move towards the mucilage. Treatment 4 served as a positive environmental control where seeds were placed on a sample of untreated raw soil to determine if microbes passively move towards the mucilage through direct contact between the seed mucilage and the soil. After 3 days of exposure to treatments, each seed was individually removed and the mucilage around the base of the hypocotyl was lightly streaked across one bacteriological and one fungal agar growth medium under sterile conditions. Isolates were sub-cultured to obtain pure culture colonies of each identified morphotype, as described in the protocol above. Morphotypes were visually compared to a reference database created from all sequenced microbes (as described in the section above).

The total number of unique microbial morphotypes was recorded in order to assess microbial richness associated with the mucilage of all seeds exposed to the four treatments. Microbial morphotype count data were analysed with the best fitting generalized linear mixed effects model using the lme4 package [30] in the R statistical environment, version 3.4.1 (R-Core-Team, 2014). Treatment type and sampling location were entered as fixed effects and *Oxalis* host species as a random effect. Residual plots did not reveal any obvious deviations from normality or homoscedasticity. A post-hoc Tukey test was used to compare estimated values between the four treatment groups.

### Seed, seedling and plant anatomy

All *Oxalis* bulb, leaf, seed and seedling material (sampled from a research collection and the wild) was fixed in Formalin-Acetic-Acid-Alcohol (FAA), dehydrated in an alcohol series and gradually infiltrated with and embedded in paraffin wax [31]. Samples were sectioned with a rotary microtome (Leitz, Germany). Sections were stained using the Safranin-Alcian-blue or Safranin-Fastgreen differential staining methods [31] and DPX glue was used to preserve these sections as permanent slides. Anatomical traits of embedded plant material were studied using a Nikon ECLIPSE E400 light microscope and photographed using a Leica MC 170 HD camera and LAS CORE software (Leica, Switzerland). Seedling germination and growth were documented using Leica M125 stereo microscope and LAS CORE software. Backgrounds of images and plant debris from preparing slides were removed using the ‘fuzzy select tool’ from Gimp 2.10.2.

Fresh *Oxalis* seeds were surface sterilized following the above-mentioned protocol, embedded and cryo-sectioned using a Leica CM1860 UV cryostat (Leica Biosystems). All seeds were harvested from the wild *Oxalis* populations growing at Stellenbosch. Sections were mounted on sterilized glass slides. Sectioning and staining were done on the same days as seed harvest. Slides were stained with LIVE/DEAD BacLight Bacterial Viability Kit (Life Technology), using 100 μL per slide. Slides were viewed using a Carl Zeiss Confocal LSM 780 Elyra S1 microscope from the Fluorescence Microscopy Unit (Central Analytical Facility) at Stellenbosch University, in order to detect the location of bacteria within seeds. Original red and green light fluorescence images have been changed to a colour-safe combination of magenta and lime-green (Fig. 3di-iii), using the ‘colour shift function’ (310° to 319°) in Inkscape 0.92.3.

## Supplementary information

**Additional file 1: Figure S1.** Seedling germination and development of recalcitrant Cape *Oxalis*, where foliar leaf development and growth is followed by delayed radicle growth. *O. clavifolia* Sond. (a) and *O. glabra* Thunb. (b) one (i), three (ii), five (iii) and 10 (iv) days after germination. All seedlings oriented with radicle pointing to bottom of figure. CT = cotyledons, FL = foliar leaf, RD = radicle.

**Additional file 2: Figure S2.** Phylogenetic consensus tree constructed with universal 16S region sequences for endophytic bacteria isolated from Cape *Oxalis* (boldface font) and representative GenBank BLAST results. Colour boxes indicate the most likely species identifications of *Oxalis* isolates. *B. megaterium* and *B. aryabhattai* that had unresolved relationships based on the consensus tree.

**Additional file 3: Figure S3.** Original red-and-green confocal staining images of cross sections of sterilized *Oxalis* seeds indicating bacteria (bright green rods) inside cavities. (i-ii) *O. hirta*, (iii) *O. pes-caprae*.

**Additional file 4: Table S1.** Properties of bacterial endophytes isolated from *Oxalis* host plants, as described in literature [57–67].

**Additional file 5: Table S2.** A key to all species names relating to Fig. 2.

## Data Availability

The datasets supporting the conclusions of this article are included within the article and its additional files (Additional file 1: Figure S1, Additional file 2: Figure S2, Additional file 3: Figure S3, Additional file 4: Table S1 and Additional file 5: Table S2).

## Abbreviations

EB: Endophytic bacteria
N- fixation: Nitrogen fixation
