# Supplementary figures and images for: Nitrogen-fixing bacteria and Oxalis – evidence for a vertically inherited bacterial symbiosis
Source: BMC Plant Biol. 2019 Oct 23;19:441. doi: 10.1186/s12870-019-2049-7 (PMC6806586; doi:10.1186/s12870-019-2049-7)

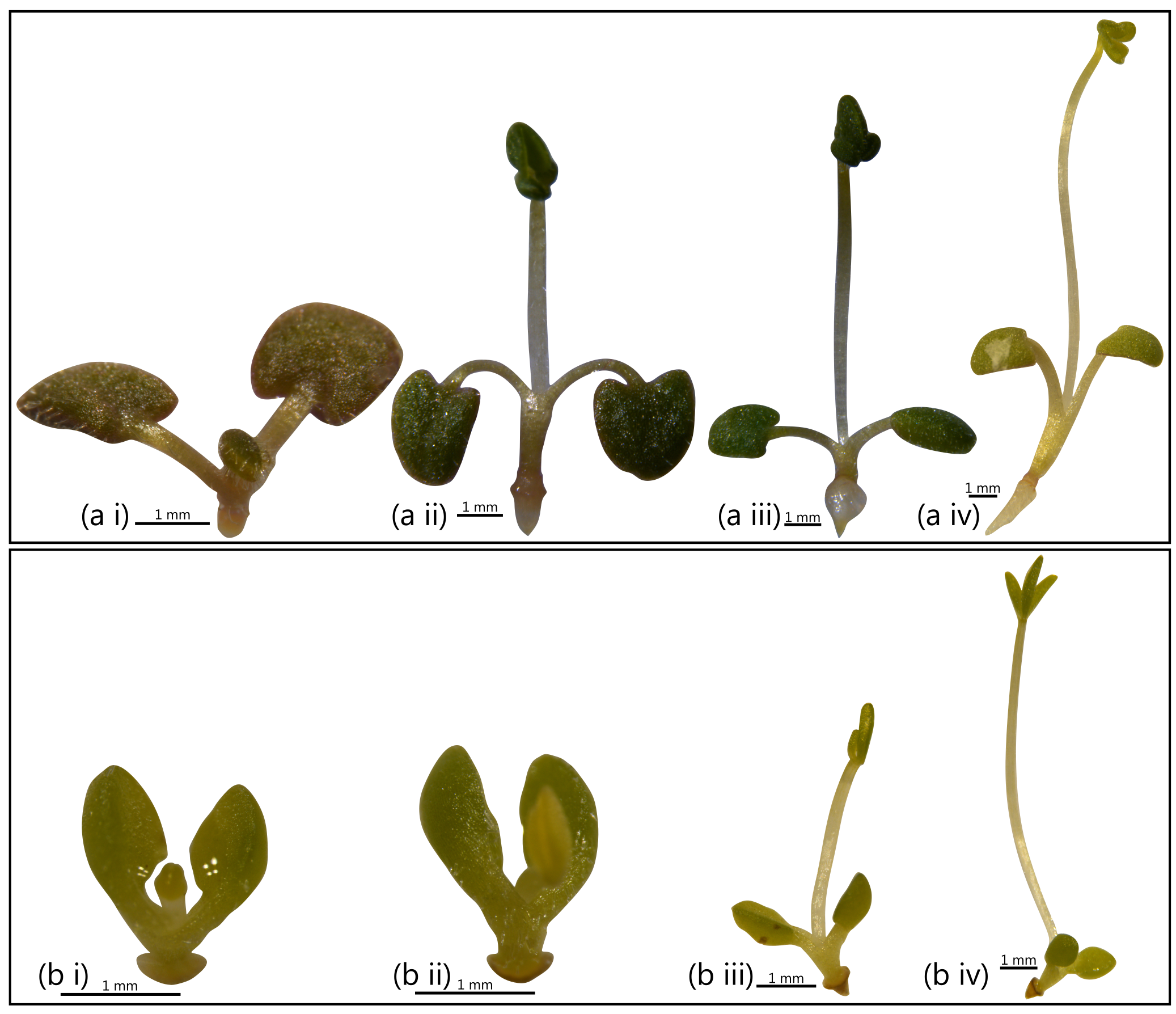

Supplement: Supplementary file 1 — Additional file 1: Figure S1. Seedling germination and development of recalcitrant Cape Oxalis, where foliar leaf development and growth is followed by delayed radicle growth. O. clavifolia Sond. (a) and O. glabra Thunb. (b) one (i), three (ii), five (iii) and 10 (iv) days after germination. All seedlings oriented with radicle pointing to bottom of figure. CT = cotyledons, FL = foliar leaf, RD = radicle. [file 12870_2019_2049_MOESM1_ESM.png]

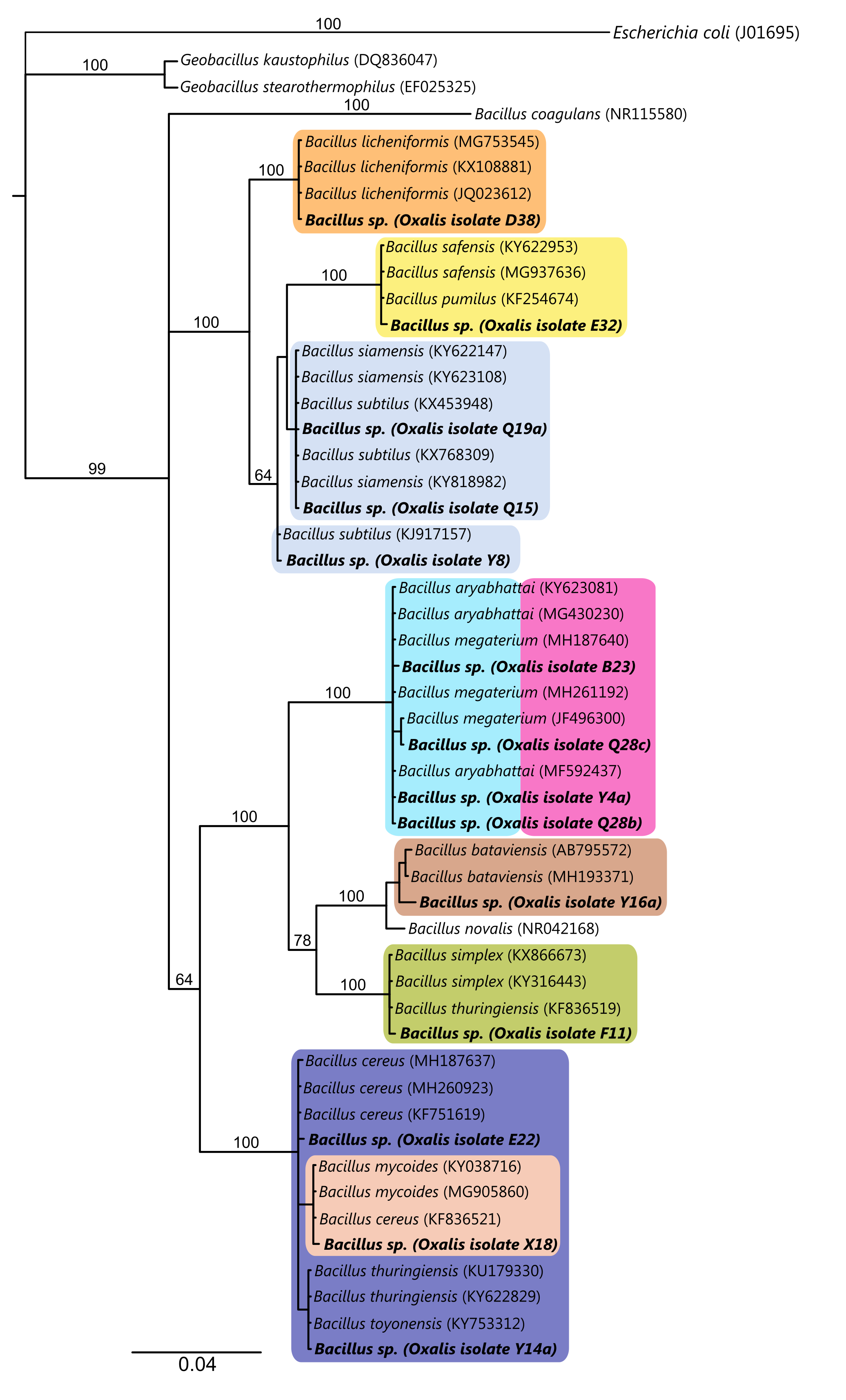

Supplement: Supplementary file 2 — Additional file 2: Figure S2. Phylogenetic consensus tree constructed with universal 16S region sequences for endophytic bacteria isolated from Cape Oxalis (boldface font) and representative GenBank BLAST results. Colour boxes indicate the most likely species identifications of Oxalis isolates. B. megaterium and B. aryabhattai that had unresolved relationships based on the consensus tree. [file 12870_2019_2049_MOESM2_ESM.png]

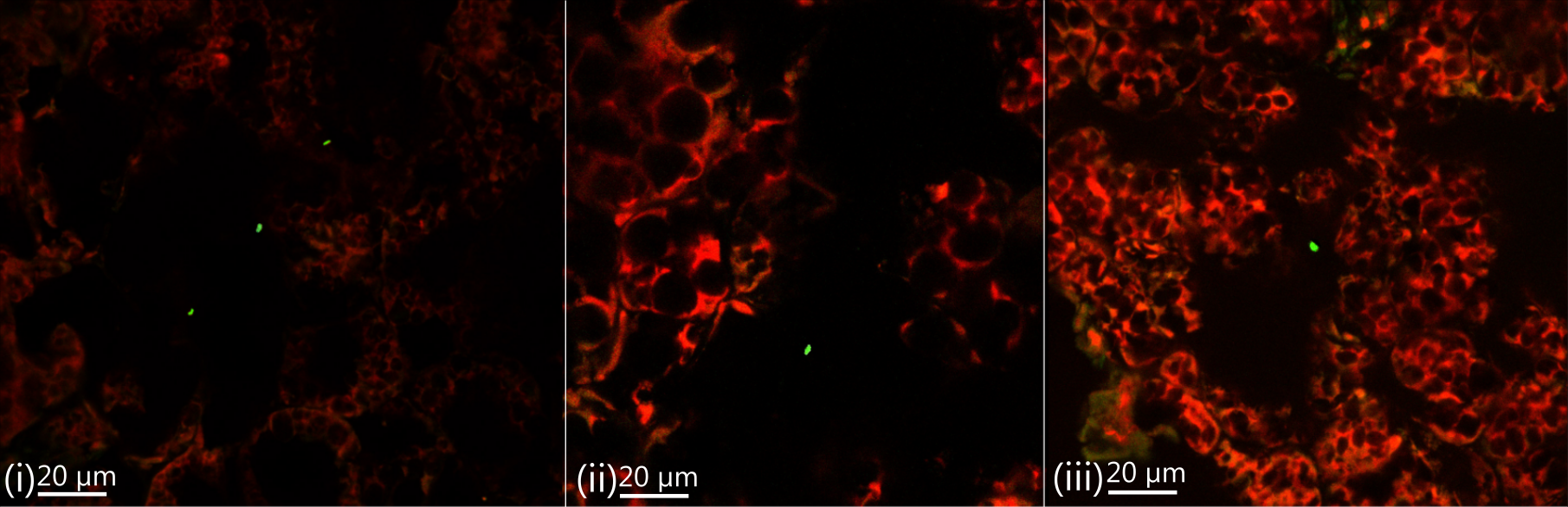

Supplement: Supplementary file 3 — Additional file 3: Figure S3. Original red-and-green confocal staining images of cross sections of sterilized Oxalis seeds indicating bacteria (bright green rods) inside cavities. (i-ii) O. hirta, (iii) O. pes-caprae. [file 12870_2019_2049_MOESM3_ESM.png]
