# Supplementary material for: Nitrogen-fixing bacteria and Oxalis – evidence for a vertically inherited bacterial symbiosis
Source: BMC Plant Biol. 2019 Oct 23;19:441. doi: 10.1186/s12870-019-2049-7 (PMC6806586; doi:10.1186/s12870-019-2049-7)
Supplement: Supplementary file 4 — Additional file 4: Table S1. Properties of bacterial endophytes isolated from Oxalis host plants, as described in literature [57–67]. [file 12870_2019_2049_MOESM4_ESM.docx]

Table S1: **Properties of bacterial endophytes isolated from *Oxalis* host plants, as described in literature.**

| Bacterial endophyte | Known beneficial traits | Oxalotrophic metabolism | References |
| --- | --- | --- | --- |
| *B. aryabhattai* | Mobilization of zinc  Various plant growth promoting mechanisms | *No* | (57) |
| *B. bataviensis* | Possible nitrogen fixation Improved availability of nitrogen | *No* | (58) |
| *B. cereus* | Nitrogen fixation  Various plant growth promoting mechanisms | *Yes* | (58), (29) |
| *B. licheniformis* | Nitrogen fixation  Phosphate solubilization | *Yes* | (60), (61) |
| *B. megaterium* | Nitrogen fixation  Phosphate solubilization | *No* | (59), (62) |
| *B. safensis* | Possible nitrogen fixation Improved availability of nitrogen | *No* | (40) |
| *B. siamensis* | Anti-fungal activity | *No* | (63) |
| *B. simplex* | Phosphate solubilization  Anti-fungal activity | *No* | (64), (65) |
| *B. subtilus* | Nitrogen fixation  Various plant growth promoting traits  Anti-pathogenic fungal activity | *Yes* | (63), (56) |
| *B. thuringiensis* | Various plant growth promoting traits  Anti-pathogenic fungal activity | *No* | (66), (67) |
