# Supplementary material for: Nitrogen-fixing bacteria and Oxalis – evidence for a vertically inherited bacterial symbiosis
Source: BMC Plant Biol. 2019 Oct 23;19:441. doi: 10.1186/s12870-019-2049-7 (PMC6806586; doi:10.1186/s12870-019-2049-7)
Supplement: Supplementary file 5 — Additional file 5: Table S2. A key to all species names relating to Fig. 2. [file 12870_2019_2049_MOESM5_ESM.docx]

Table S2: **A key to all species names relating to Figure 2.**

| Figure label | | *Oxalis* species | Germination strategy |
| --- | --- | --- | --- |
| *a)* | *i-iii* | *O. hirta* L. | Recalcitrant |
| *b)* | *i* | *O. pulchella* Jacq. | Dormant |
|  | *ii* | *O. camelopardalis* Salter | Recalcitrant |
|  | *iii* | *O. foveolata* Turcz. | Dormant |
| *c)* | *i* | *O. cf. purpurea* | Dormant |
|  | *ii* | *O. cf. hirta* | Recalcitrant |
|  | *iii* | *O. cf. purpurea* | Dormant |
|  | *iv* | *O. cf. hirta* | Recalcitrant |
| *d)* | *i* | *O. grammophylla* Salter | Recalcitrant |
|  | *ii* | *O. xantha* Salter | Recalcitrant |
|  | *iii* | *O. cf. pardales* | Recalcitrant |
| *e)* | *i-ii* | *O. hirta* | Recalcitrant |
|  | *iii* | *O. pes-caprae* | Dormant |
